# How Do Discrepancies between Victimization and Rejection Expectations in Gay and Bisexual Men Relate to Mental Health Problems?

**DOI:** 10.3389/fpsyg.2017.00857

**Published:** 2017-05-24

**Authors:** Frank A. Sattler, Hanna Christiansen

**Affiliations:** Department of Clinical Child and Adolescent Psychology, Philipps University of MarburgMarburg, Germany

**Keywords:** rejection expectations, victimization, expectation violations, mental health, gay and bisexual men

## Abstract

**Introduction:** Victimization and rejection expectations predict mental health problems in gay and bisexual men. Furthermore, it was shown that victimization predicts rejection expectations. Nevertheless, the levels of these two variables do not necessarily correspond as indicated by low inter-correlations, resulting in the question “How do discrepancies in the two variables relate to mental health problems?” This study tests if non-corresponding levels of victimization and rejection expectations in gay and bisexual men relate to mental health problems differently than corresponding levels of victimization and rejection expectations. It furthermore tests for linear and curvilinear relationships between victimization, rejection expectations, and mental health problems.

**Methods:** Data from *N* = 1423 gay and bisexual men were obtained online. Victimization and rejection expectations were tested for discrepant values (differing 0.5 SD or more) and those that were in agreement (differing less than 0.5): 33.7% of participants were in agreement, 33.0% reported higher rejection expectations than victimization, and 33.3% v.v. Then, a polynomial regression and a surface analysis were conducted.

**Results:** Discrepant values in victimization and rejection expectations or the direction of the discrepancy did not relevantly predict mental health problems. Findings indicate that victimization and rejection expectations predict mental health problems linearly as well as convexly (upward curving) in gay and bisexual men.

**Discussion:** This study replicates findings that gay and bisexual men with more experiences of victimization and rejection expectations demonstrated more mental health problems. Furthermore, this study is the first one to find a convex relationship between these predictors and mental health problems, implicating that disproportionally high mental health problems exist in those gay and bisexual men with high levels of victimization and rejection expectations. On the other hand, discrepancies between these two variables do not predict mental health problems. Future studies are needed to test for replication of our findings.

## Introduction

According to minority stress theory, a number of minority stressors lead to mental health problems in gay and bisexual men, resulting in mental health disparities between gay and bisexual men in comparison to heterosexual men (Meyer, 2003; King et al., 2008). Minority stressors faced by gay and bisexual men include gay-related victimization, discrimination, rejection expectations (chronic expectations of gay-related rejection), internalized homonegativity (or internalized homophobia), boyhood gender non-conformity, and masculine standards (Pachankis, 2015). It is proposed that these stressors lead to a higher number of mental health problems as other non-minority specific stressors (e.g., work stress or marital stress) would do, too. Among the minority stressors with the broadest empirical evidence are gay-related victimization (i.e., victimization of gay and bisexual men due to their sexual orientation) and rejection expectations (i.e., expectation of being a target of victimization in the future). Numerous cross-sectional studies and some longitudinal ones have found that these minority stressors linearly predict gay and bisexual men’s mental health problems (Feinstein et al., 2012; Burton et al., 2013; Pachankis et al., 2014a; Eldahan et al., 2016; Sattler et al., 2016). Up to date no studies exist that tested for a curvilinear (squared) relationship between these variables. The knowledge is thus very limited on how both variables might interact with one another.

Furthermore, it was proposed that minority stressors are not independent from each other but that gay-related victimization (from now on abbreviated as victimization) predicts expectations such as rejection expectations (Fredriksen-Goldsen et al., 2014). Indeed, cross-sectionally it was demonstrated that victimization predicted rejection expectations in lesbians and gay men (Feinstein et al., 2012). Nevertheless, the level of rejection expectations does not necessarily correspond to the level of victimization in each gay or bisexual man as shown by studies reporting low associations (*r* = 0.20 to 0.29) between the two variables (Pachankis et al., 2014b; Sattler et al., 2016). There are two possible scenarios: (1) an individual may expect to be rejected although they have been victimized in the past to a non-correspondingly low degree or, (2) an individual may expect very little rejection despite having been victimized in the past to a non-correspondingly high degree. In both cases, an expectation violation is prevalent; or in other words discrepancies exist between victimization and rejection expectations.

The primary goal of the study is to empirically investigate these expectation violations. As implied by earlier studies, we therefore hypothesize that we will find a linear relationship between victimization, rejection expectations, and mental health (hypothesis 1). Furthermore, we were interested in whether the relation between victimization, rejection expectations, and mental health problems is best described as merely linear or if an interaction exists. We therefore wanted to test whether differing levels of victimization and rejection expectations will predict differing levels of mental health problems, in addition to the predictions depicted in hypothesis 1, and whether victimization and rejection expectations predict mental health problems curvilinearly (squaredly).

## Materials and Methods

### Data Collection

The survey was conducted online in a number of German web sites for gay and bisexual men as well as mailing lists for students and employees of the Philipps University of Marburg (PUM). This study was carried out in accordance with the recommendations of the Ethics Committee of the Psychological Faculty of the Philipps University of Marburg (PUM) with online informed consent from all subjects. All subjects gave online informed consent in accordance with the Declaration of Helsinki. The study was approved by the Ethics Committee of the Psychological Faculty of the PUM.

### Participants

In total, *N* = 1737 gay and bisexual men participated in the survey in 2014. Participants who indicated that they were younger than 18 years (*n* = 3), older than 80 years (*n* = 18), or who did not complete the questionnaire (*n* = 293) were excluded from analyses. The final sample thus consisted of *N* = 1423 gay and bisexual men. Of these men, *n* = 1308 (91.9%) defined as gay and *n* = 115 (8.1%) defined as bisexual. Furthermore, *n* = 146 (10.3%) were immigrants or had at least one immigrant parent. The relationship status was as follows: *n* = 688 (48.3%) gay and bisexual men were in a relationship with a man; *n* = 158 (11.1% of the total sample) of them were in a civil union. Furthermore, *n* = 50 (3.5%) were in a relationship with a woman; *n* = 32 (2.2% of the total sample) of them were married. Finally, *n* = 691 (48.6%) were single. The education levels were as follows: *n* = 3 (0.2%) no school degree, *n* = 57 (4.0%) junior high school degree, *n* = 193 (13.6%) middle high school degree, *n* = 420 (29.5%) senior high school degree, *n* = 624 (43.9%) university degree, and *n* = 126 (8.9%) doctoral degree.

### Measures

#### Victimization

It was assessed with five items of the victimization scale by Herek and Berrill (1992). The items asked for victimization since the age of 16 years. While the original scale used a three-point response format (from 1 = *never* to 3 = *two or more*), we used an amplified four-point response format (from 1 = *never* to 4 = *three times or more*). Cronbach’s alpha of the scale was 0.76 in the present study.

#### Rejection Expectations

It was assessed with three items of the Gay-Related Rejection Sensitivity Scale (Pachankis et al., 2008). The participants read three short texts on potentially homonegative situations and reported whether they would feel discriminated upon in these situations due to their sexual orientation. A five-point response format was used (from 1 = *strongly disagree* to 5 = *strongly agree*). Cronbach’s alpha of the scale was 0.65 in the present study. Due to the Cronbach’s alpha that was between the thresholds of questionable (0.60) and sufficient (0.70), a principal component factor analysis (κ = 4; number of iterations = 1000) was applied to test the factorial validity of the rejection expectations scale. Only one component with an eigenvalue > 1 was extracted, thereby explaining 58.3% of the variance. All items loaded on the component between *λ* = 0.71 and 0.81.

#### Mental Health

The problems were assessed with 27 items of the Brief Symptom Inventory (Franke, 2000). The items assessed symptoms of somatization, obsessive-compulsive disorder, interpersonal sensitivity, depression, anxiety, hostility, phobic anxiety, paranoid ideation, and psychoticism. A five-point response format was used (from 1 = *not at all* to 5 = *extremely*). The scale’s Cronbach’s alpha was 0.95 in the present study.

### Data Analysis

For data analysis, zero-order Pearson’s correlations between the main constructs were computed. Scores for rejection expectations and victimization were z-standardized. Then, a polynomial regression with response surface analysis was conducted using the approach described by Shanock et al. (2010) that includes the following steps: first, descriptive information was provided about the occurrence of discrepancies within the variables victimization and rejection expectations. Thereby, any participant with the two scores differing half a standard deviation or more were considered to have discrepant values (Shanock et al., 2010), while the rest was considered to have agreeing values for the two constructs. Second, a polynomial regression was conducted in IBM Statistics SPSS 22 and the surface values were conducted afterward. Thereby, the predictors were centered around the midpoint of their respective scales (Shanock et al., 2010). Then, the following variables were computed: the square of the centered variable victimization, the square of the centered variable rejection expectations, and the cross-product of both centered variables. Afterward, a polynomial regression was conducted using the centered predictor variables, the squared variables, and the cross-product variable as predictors. Mental health problems were used as the criterion. Third, the surface values were interpreted.

## Results

### Descriptive Data Analysis

Victimization was positively inter-correlated with rejection expectations (*r* = 0.25, *p* < 0.001). Moreover, mental health problems were positively associated with victimization and rejection expectations (*r* = 0.31 to 0.34, *p* < 0.001). See **Table 1** for further details.

**Table 1:** Intercorrelations of the scales.

Scale	1	2	3
(1) Victimization			
(2) Rejection expectations	0.25^∗∗∗^		
(3) Mental health problems	0.34^∗∗∗^	0.31^∗∗∗^	
Mean (SD)	1.59 (0.64)	2.87 (0.98)	1.60 (0.60)
Min–Max	1–4	1–5	1–4.19


*SD, standard deviation; Min, minimum; Max, maximum. ^∗∗∗^p < 0.001*.

### Polynomial Regression with Response Surface Analysis

#### Step 1: Descriptive Information on Discrepancies

Data suggests the values in victimization and rejection expectations were in agreement for 33.7% of participants (meaning that they differed less than 0.5 SD), while 33.0% reported higher rejection expectations than victimization, and 33.3% reported higher victimization than rejection expectations (see **Table 2**). Since 66.3% of the predictor variables showed discrepant values, it is meaningful to use a polynomial regression for further data analysis.

**Table 2:** Agreement between victimization and rejection expectations.

Agreement groups	Percentage	Number	Mean (SD) V	Mean (SD) RE
RE more than V	33.0	470	1.27 (0.31)	3.66 (0.61)
In agreement	33.7	479	1.52 (0.48)	2.81 (0.72)
V more than RE	33.3	474	1.98 (0.79)	2.14 (0.92)


*SD, standard deviation; V, victimization; RE, rejection expectations*.

#### Step 2: Polynomial Regression and Surface Values

**FIGURE 1:**
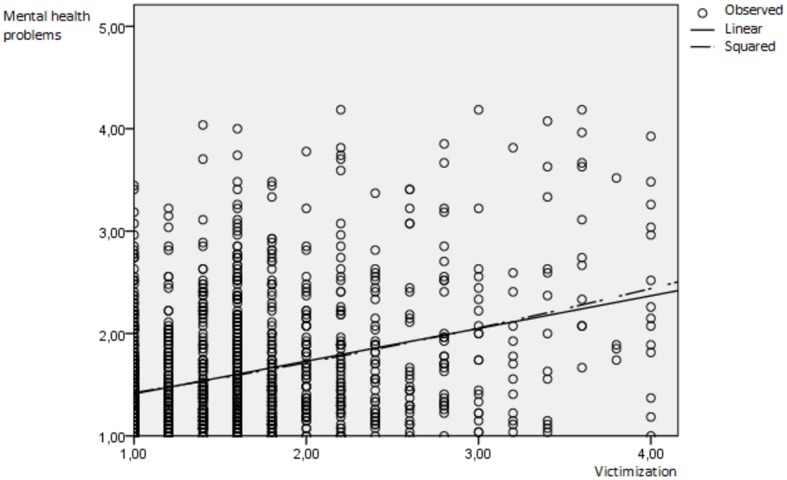
**Linear and convex prediction of mental health problems by victimization.** Observed = observed value, linear = linear line of parameter estimation, squared = squared (convex) line of parameter estimation.

**FIGURE 2:**
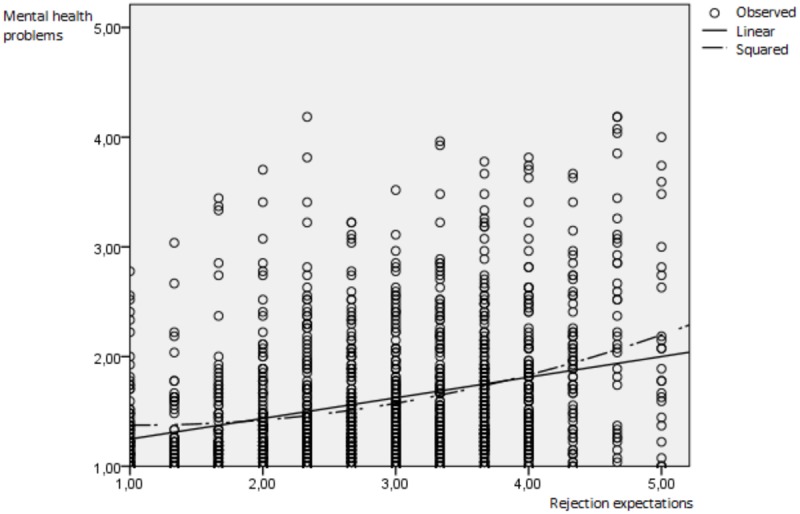
**Linear and convex prediction of mental health problems by rejection expectations.** Observed = observed value, linear = linear line of parameter estimation, squared = squared (convex) line of parameter estimation.

The centered variable victimization (β = 0.25, *p* < 0.001), as well as the centered variable rejection expectations (β = 0.18, *p* < 0.001) significantly predicted mental health problems (see **Table 3**). Furthermore, the squared variable rejection expectations predicted mental health problems (β = 0.04, *p* < 0.01), while victimization squared did not predict mental health problems (β = 0.01, *p* > 0.05). The linear as well as the squared relationships are displayed in **Figures 1, 2**. Furthermore, the cross-product of victimization centered and rejection expectations centered significantly predicted mental health problems (β = 0.05, *p* < 0.05). However, since the predictions by rejection expectations squared and the cross-product were below β < 0.10, we interpret them as not relevant in order to not over-interpret our findings (Nathans et al., 2012). All predictors included in the polynomial regression explained 17.6% of the variance in mental health problems.

**Table 3:** Discrepancy between victimization and rejection expectations as predictor of mental health problems.

Predictor	β (SE)
Victimization	0.25 (0.02)^∗∗∗^
Rejection expectations	0.18 (0.02)^∗∗∗^
Victimization squared	0.01 (0.03)
Rejection expectations squared	0.04 (0.01)^∗∗^
Victimization × rejection expectations	0.05 (0.03)^∗^
*Surface test*	
*a_1_*	0.43^∗∗∗^
*a_2_*	0.10^∗∗∗^
*a_3_*	0.07^∗^
*a_4_*	0.02


*Victimization and rejection expectations are centered around the midpoint of the respective scales, β, unstandardized beta weight; SE, standard error; a_1_, slope of the line of perfect agreement; a_2_, curvature along the line of perfect agreement; a_3_, slope of the line of incongruence; a_4_, curvature of the line of incongruence. ^∗^p < 0.05, ^∗∗^p < 0.01, ^∗∗∗^p < 0.001*.

In addition, the surface values were predicted for the polynomial regression: these include the slope of the line of perfect agreement (when victimization and rejection expectations are in agreement) a_1_, the curvature along the line of perfect agreement (when a squared relationship exists) a_2_, the slope of the line of incongruence (when discrepancies between victimization and rejection expectations exist) a_3_, as well as the curvature of the line of incongruence (when a squared relationship exists) a_4_. In the current polynomial regression, a_1_ (β = 0.43, *p* < 0.001), a_2_ (β = 0.10, *p* < 0.001), and a_3_ (β = 0.07, *p* < 0.05) proved to be significant, while a_4_ (β = 0.02, *p* > 0.05) was not significant (compare **Table 3**).

#### Step 3: Interpretation of the Surface Values

Since a_1_ was significant (β = 0.43, *p* < 0.001), there is a linear (additive) relationship between victimization, rejection expectations, and the outcome. Consequently, mental health problems are predicted positively by agreeing levels of victimization and rejection expectations. Hypothesis 1 was therefore confirmed.

A significant a_2_ (β = 0.10, *p* < 0.001) indicates that there is a non-linear slope of the line of perfect agreement. This means that the line has a convex (upward curving) surface, indicating that mental health increases to a steeper degree by increasing levels of agreeing victimization and rejection expectations. A squared relationship between these variables, was therefore not found. Both the linear as well as the squared predictions of mental health problems are depicted separately for both predictors in **Figures 1, 2**.

Since a_3_ was significant (β = 0.07, *p* < 0.05), the direction of the discrepancy is related to the outcome: mental health problems are higher when victimization exceeds rejection expectations. However, a_3_ was below a level of β > 0.10 and is thus no relevant predictor of mental health problems. Furthermore, a non-significant a_4_ (β = 0.02, *p* > 0.05) indicates that a stronger discrepancy does not predict a higher level of mental health problems. Therefore, no squared relationship between a discrepancy and mental health problems exists.

In summary, mental health problems were predicted linearly and furthermore convexly by agreeing levels of victimization and rejection expectations. No relevant prediction was found for discrepant values or the direction of the discrepancy.

## Discussion

This study is the first one to investigate if discrepancies between victimization and rejection expectations reflect on the mental health of sexual minorities.

In a sample of *N* = 1423 gay and bisexual German men, we found that agreeing levels of victimization and rejection expectations predicted mental health problems linearly as well as convexly (squaredly). Our study therefore replicates a great number of studies that found evidence of victimization and rejection expectations to predict mental health problems linearly (Frisell et al., 2010; Feinstein et al., 2012; McLaughlin et al., 2012; Burton et al., 2013; Eaton, 2014; Sattler et al., 2016). On the other hand, the findings of the squared relationship are unique: to the author’s knowledge we are the first ones to demonstrate that when victimization and rejection expectations are both high, disproportionately higher levels of mental health problems are found than expected by both predictors separately (compare **Figures 1, 2**). Note that when victimization squared and rejection expectations squared were used as individual predictors in the polynomial regression, only rejection expectations squared showed a significant prediction of mental health problems. It is possible that gay and bisexual men are overloaded by a high number of victimization events and especially by a high level of rejection expectations leading to a stronger increase in mental health problems. Another explanatory model is that gay and bisexual men with higher levels of mental health problems may overestimate their level of victimization and rejection expectations as found in individuals with depression due to their tendency for biased attention, processing, thoughts, and memory (Disner et al., 2011). Future research is needed to replicate the findings as well as to test possible explanatory models.

Furthermore, we did not find that discrepant values in victimization and rejection expectations predicted mental health problems at a relevant level. While a significant prediction was found when victimization was higher than rejection expectations, the size of the prediction was at an irrelevant level. This implicates that it is slightly adaptive for gay and bisexual men to have a level of rejection expectations that is higher than or corresponding to the level of experienced victimization. A possible explanation could be that rejection expectations help gay and bisexual men to process victimization. However, since this relationship was very low, we interpret it as not externally relevant.

Moreover, longitudinal and experimental data would be especially useful in determining the direction of the prediction between rejection expectations and mental health.

Limitations of the study include that a cross-sectional approach was used. It is therefore possible that the predictions are inversed, i.e., mental health problems predicting higher victimization. A further limitation was that the used scales had not been previously validated and that the Cronbach’s alpha coefficient of rejection expectation was between questionable (0.60) and sufficient (0.70). However, a *post hoc* factorial analysis confirmed a one-factor solution for this scale. Thereby, factorial validity of the scale could be established. Nevertheless, type-II errors derived from this scale are still more likely in the current study and the correlations between rejection expectations and victimization as well as rejection expectations and mental health problems are likely to be underestimated.

## Conclusion

This study provides the first evidence for a curvilinear (upward curving) relationship between victimization, rejection expectations, and mental health problems. It also replicates findings documenting a linear relationship between victimization, rejection expectations, and mental health problems. Furthermore, discrepancies in victimization and rejection expectations are not associated with mental health problems.

## Author Contributions

All authors listed, have made substantial, direct and intellectual contribution to the work, and approved it for publication.

## Conflict of Interest Statement

The authors declare that the research was conducted in the absence of any commercial or financial relationships that could be construed as a potential conflict of interest.
